# Occupational Complexity and Risk of Parkinson's Disease

**DOI:** 10.1371/journal.pone.0106676

**Published:** 2014-09-08

**Authors:** Elise G. Valdés, Ross Andel, Johanna Sieurin, Adina L. Feldman, Jerri D. Edwards, Niklas Långström, Margaret Gatz, Karin Wirdefeldt

**Affiliations:** 1 University of South Florida, School of Aging Studies, Tampa, Florida, United States of America; 2 St. Anne's University Hospital, International Research Center, Brno, Czech Republic; 3 Karolinska Institutet, Department of Medical Epidemiology and Biostatistics, Stockholm, Sweden; 4 University of Southern California, Department of Psychology, Los Angeles, California, United States of America; 5 Karolinska Institutet, Department of Clinical Neurosciences, Stockholm, Sweden; University of Toronto, Canada

## Abstract

**Background:**

The etiology of Parkinson's disease (PD) remains unclear, and environmental risk-factors such as occupation have attracted interest.

**Objective:**

The goal was to investigate occupational complexity in relation to PD.

**Methods:**

We conducted a population-based cohort study based on the Swedish Twin Registry that included 28,778 twins born between 1886 and 1950. We identified 433 PD cases during the study period. Data on occupation were collected from either the 1970 or 1980 Swedish census, and occupational complexity was assessed via a job exposure matrix. Cox proportional hazard regression analyses with age as the underlying time scale were used to assess PD risk as a function of the three domains of occupational complexity: data, people, and things. Sex and smoking were included as covariates. Analyses stratified by twin pair were conducted to test for confounding by familial factors.

**Results:**

High occupational complexity with data and people was associated with increased risk overall (Hazard Ratio [HR] = 1.07, 95% confidence interval [CI] 1.02–1.14, and HR = 1.10, 95% CI 1.01–1.21, respectively), and in men (HR = 1.08, 95% CI 1.01–1.16, and HR = 1.15, 95% CI 1.03–1.28, respectively). Complexity with things was not associated with risk of PD. When the analyses were stratified by twin pair, the HRs for occupational complexity with data and people were attenuated in men.

**Conclusions:**

High complexity of work with data and people is related to increased risk of PD, particularly in men. The attenuation of risk observed in the twin pair-stratified analyses suggests that the association may partly be explained by familial factors, such as inherited traits contributing to occupational selection or other factors shared by twins.

## Introduction

Parkinson's disease (PD) is the second most common neurodegenerative disorder in adults aged 60 and older [1]. With the continued growth of the older adult population, PD prevalence in people over age 50 is expected to double from between 4.1 and 4.6 million in 2005 to between 8.7 and 9.3 million by 2030 in 15 of the world's most populous nations [2]. Designing effective prevention and treatment options is directly linked to understanding risk factors for PD, making risk factor research essential. Etiology of PD is likely influenced by both environmental and genetic factors. Data from several studies suggest that environmental factors may be more important than genetic factors in risk of PD [3]. Moreover, environmental exposures are often potentially modifiable and can be especially useful in disease prevention. Thus, it is unsurprising that numerous aspects of the environment have already been investigated with evidence accumulating that occupation-related exposures may be important [4]–[6].

Notably, while Alzheimer's disease research unequivocally indicates occupations reflecting low socioeconomic status [7] or low complexity of work [8] as risk factors, the picture is less clear, if not reversed, in research with PD. Specifically, higher education [4], [9] and higher-status occupations [4]–[6] have been associated with a higher risk of PD or the results were null [10]. These findings are not well understood. Both education and occupational status may differentiate individuals based on intellectual engagement in various aspects of life. Occupational complexity is another, more refined way to measure intellectual engagement at work.

The concept of occupational complexity [11] captures cognitive effort required to perform a certain job, measured along the dimensions of complexity of work with data, people, and things. High occupational complexity is sometimes (but not always) reflected in higher education and/or higher occupational status. Building on research pointing to the association between high education level, high occupational status and greater risk of PD, we investigated the role of occupational complexity in risk of PD. Further, we used the unique opportunity to use data from the Swedish Twin Registry to assess directly whether any observed findings may be explained by familial factors shared by twins.

We hypothesized that higher occupational complexity would be associated with increased risk of PD. We expected these results to exist for complexity of work with data and people in particular, which are often found to relate to health outcomes more strongly than complexity with things. Finally, we expected that any results would be attenuated in the twin pair analyses compared to the analyses with the entire sample, suggesting that familial factors such as inherited traits contributing to occupational selection or other factors shared by twins potentially underlie the results.

## Materials and Methods

### Study population

The study is in compliance with the Swedish Act concerning the Ethical Review of Research Involving Humans. The study was approved by the regional Ethics Committee in Stockholm. Patient information was de-identified prior to analysis. As this was a register-based study, it was not possible to obtain written consent for all study participants.

The Swedish Twin Registry (STR) [12] is the largest population-based twin registry in the world, containing information on Swedish twins born 1886 and onwards. In the 1960's, same-sexed twins born 1886–1925 were contacted with several questionnaires regarding demographic, medical and life-style factors. In 1973, same-sexed twins born 1926–1958 were contacted with a similar questionnaire.

Between 1965 and 1990, national censuses were carried out every fifth year by Statistics Sweden, collecting information on current occupation, income, and type and size of habitation. These censuses have been linked to the STR through the unique 10-digit personal identification number assigned to all Swedish residents.

For the current study, we considered same-sexed twins of the STR born 1886–1950. Information from the 1970 and 1980 censuses were used for those born between 1886–1925 and 1926–1950, respectively. Twins born 1886–1925 were included in the study population if they completed the first questionnaire in 1961 and were alive and without PD, according to the National Patient Register (NPR) and the Cause of Death Register (CDR), during the 1970 census. Twins born 1926–1950 were included if they completed the questionnaire in 1973, and were at least 30 years old, alive and without PD, according to the National Patient Register (NPR) and the Cause of Death Register (CDR), during the 1980 census.

The original cohort contained 49,585 individuals. Of these, 2,925 individuals were excluded because they died prior to the study period, were lost to follow-up, had PD before baseline, or it was not possible to link their data. Further, 17,882 individuals were excluded due to lack of complete data on the occupation or smoking variables. Of the people with missing occupational data, 75% were women. Therefore, sex was included as a covariate in our analyses. The primary reason for lack of smoking data was failure of the participants to return a completed survey. The final study population consisted of 28,778 twins with 10,899 complete twin pairs.

### Occupational Complexity

Information on occupation was obtained from the question on current occupation in the 1970 or 1980 Swedish census. For twins born 1886–1925, occupational data were obtained from the 1970 Census, when the respondents were between 45 and 82 years old (*M* = 54.3 years, *SD* = 6.1). For twins born 1926–1950 occupational data were obtained from the 1980 Census, when the respondents were between 30 and 55 years old (*M* = 41.3 years, *SD* = 7.1). Occupations were coded on a 3-digit level, based on the Nordic Standard Occupational Classification of 1965 (‘Nordisk yrkesklassificering’; NYK), adapted from the International Standard Classifications [13].

A job exposure matrix(JEM) was used to assess occupational complexity. Occupational complexity scores reflect those created in the fourth edition of the *Dictionary of Occupational Titles* [14] derived through extensive site observation of over 12,000 occupations by qualified job analysts at multiple sites throughout the US [14]. Each occupation was rated regarding complexity of work with data, people, and things. See Table 1 for a description of the characteristics of work performed at each level of complexity.

**Table 1:** Description of the type of task associated with each level of complexity.

Score range	DATA	Score range	PEOPLE	Score range	THINGS
5.6–6.0	Synthesizing	7.6–8.0	Mentoring	6.6–7.0	Setting up
4.6–5.5	Coordinating	6.6–7.5	Negotiating	5.6–6.5	Precision Working
3.6–4.5	Analyzing	5.6–6.5	Instructing	4.6–5.5	Operating
2.6–3.5	Compiling	4.6–5.5	Supervising	3.6–4.5	Driving
1.6–2.5	Computing	3.6–4.5	Diverting	2.6–3.5	Manipulating
0.6–1.5	Copying	2.6–3.5	Persuading	1.6–2.5	Tending
0.0–0.5	Comparing	1.6–2.5	Speaking/Signaling	0.6–1.5	Feeding/Offbearing
		0.6–1.5	Serving	0.0–0.5	Handling
		0.0–0.5	Taking instructions		

Note. Shows the reverse scored range for the complexity levels and descriptions. Adapted from Ross and colleagues 2005 [8].

A study using a large sample of the US labor force in 1971 was conducted to assess inter-rater agreement of the initial ratings across occupations [15]. Kappa reliability estimates were 0.85 for complexity of work with data, 0.87 for complexity of work with people, and 0.46 for complexity of work with things [15]. Scores range from 0.0 to 6.0 for complexity of work with data, 0.0 to 8.0 for complexity of work with people, and 0.0 to 7.0 for complexity of work with things, with lower scores reflecting higher complexity. To make the results more easily interpretable, we reverse scored the complexity variables so a higher score reflects higher complexity (Table 1). For example, physicists are relatively high in complexity with data, while lawyers are high in complexity with people, and precision tool makers are high in complexity with things. In order to address the positively skewed distribution of complexity of work with people, this variable was log transformed.

To enable application of the JEM to Swedish occupational data, two independent raters, one in Sweden and the other in the United States, matched all the occupational categories from the 1970 and 1980 Swedish Census to corresponding categories from the 1970 US Census. The raters initially agreed on 90% of the matches and the differences were discussed by the raters until they were resolved. Details regarding this procedure are published elsewhere [8].

### Smoking Status

Due to previous research showing the importance of smoking as a protective factor in PD [9], smoking status was included as a covariate to control for potential confounding. Information was taken from the questionnaires sent in 1967 and 1970 for twins born 1886–1925 and in 1973 for twins born 1926–1950. Smoking status was categorized as smoker vs. not smoker at the time of the questionnaire.

### Parkinson's disease ascertainment

Incident cases of PD were identified through cross-linkage between the STR, the National Patient Register [16] (NPR) and the Cause of Death Register [17] (CDR). The NPR was initiated by the National Board of Health and Welfare in 1964 and contains information on hospital discharge diagnoses from all hospitals in Sweden (mandatory reporting for all public and private hospitals). The register was gradually expanded and full national coverage for somatic disorders was obtained in 1987 [18]. Since 2001, the NPR also includes outpatient consultations with specialist physicians at hospital-based clinics. For the present study, data were available through 2009. The CDR contains information on all deaths of Swedish residents since 1961, including information on underlying and contributory causes of death. Causes of death data were available through 2008 and information on vital status through 2009. Diagnoses were coded according to the International Classification of Diseases (ICD) in both the NPR and the CDR.

Cases were defined as PD if there was a PD diagnosis in the NPR or the CDR. Both primary and secondary diagnoses, as well as underlying and contributory causes of death, were considered. Date of diagnosis was defined as the first date of diagnosis in the NPR, or the date of death in the CDR for those cases only identified at death. ICD codes used to define PD were: 342.00 (ICD-8); 332.0 (ICD-9); G20 (ICD-10). PD diagnoses in the inpatient register and the CDR have been validated against diagnoses based on clinical examinations: The positive predictive value(PPV) for PD diagnoses was 70.8% in the in-patient register and 66.7% in the CDR; the sensitivity was 83% in the two combined [19].

### Statistical Methods

The study population was followed from January 1^st^ 1971, for those born 1886–1925 or January 1^st^ 1981, for those born 1926–1950, until ascertainment of disease diagnosis, death, or December 31^st^ 2009, whichever came first. Incidence rates (IR) of PD were calculated as events divided by time-at-risk, per 100,000 person years(PYR) with 95% confidence intervals (CI) assuming a Poisson distribution.

We used two approaches to data analysis. In the first approach (Model 1) the association between occupational complexity and PD was modeled using Cox proportional hazards regression yielding hazard ratios (HR) with a 95% CI. Robust standard errors were used to adjust for any within-pair correlation and age was used as the underlying time scale in order to account for both age and follow-up interval within the model. We included sex and smoking as covariates in the model. We analyzed all individuals, as well as men and women separately. Occupational complexity was analyzed as a continuous variable.

In the second approach (Model 2) we conducted a twin pair analysis using a stratified Cox proportional hazards regression, in which each stratum comprised a twin pair. This method allows for familial factors (genetic and early-environmental) shared by twins to be controlled for as part of the design. Thus, if the results are attenuated compared to the un-stratified design it would suggest familial factors account for part of the association.

Finally, we conducted sensitivity analyses to examine differences by cohort, education, length of time in study and between people who were diagnosed while alive vs at death. Data analyses were performed with SAS software, Version 9.2 of the SAS System for Windows [20].

## Results

Demographic characteristics can be found in Table 2. We identified 433 incident cases of PD, 262 men and 171 women. The overall incidence rate of PD for the cohort was 56.0/100,000 person/years (PYR; 95% CI 51.0–61.5) which is similar to other studies [21]. Incidence rates were nearly undetectable until age 65 when, as expected, they started to rise exponentially and reached a level of 135.9/100,000 PYR in the oldest age group (90+ years), which is somewhat lower than other European studies [21].

**Table 2:** Characteristics of the study population.

			Non-Parkinson's Disease (N = 28,345)			Parkinson's Disease (N = 433)		
	Min	Max	Mean	SD	%	Mean	SD	%
**Background Characteristics**								
Age at baseline, years	30.00	82.00	46.06	9.23	.	51.92	7.42	.
Time to end of follow-up, years	0.08	39.00	26.93	7.41	.	22.57	7.46	.
Women	.	.	.	.	46.36	.	.	39.49
Current smoker (baseline)	.	.	.	.	39.04	.	.	21.71
**Occupational Complexity**								
Data	0.00	6.00	2.82	1.67	.	2.95	1.66	.
People	0.00	8.00	1.65	1.59	.	1.71	1.66	.
Things	0.00	6.80	2.83	2.24	.	2.74	2.31	.

In the first approach (see Table 3, Model 1), analyses indicated an association between higher occupational complexity of work with data and higher risk of PD, such that each additional level of complexity was related to a 7% increase in risk, or an increase in risk by 42% between the lowest and the highest level of complexity with data. There was a trend suggesting higher occupational complexity with people was associated with a 10% increase in risk of PD but this was not significant (p = .05). When the analyses were stratified by sex, occupational complexity with data and people were risk factors in men but not women (*p*'s>.05). No significant relationships were found between occupational complexity with things and risk of PD (*p*'s>.05).

**Table 3:** Age-adjusted hazard ratios and 95% CI by occupational complexity (continuous) for risk of PD, sex and smoking included as covariates.

		Model 1: Entire Cohort			Model 1 with Education		Model 2: Twin Pairs		
Variable		N	HR	95% CI	HR	95% CI	N	HR	95% CI
Data	***All***	433	1.07*	1.01–1.13	1.06	1.00–1.12	345	1.07	0.93–1.22
	*Men*	262	1.07*	1.00–1.15	1.07	0.99–1.15	226	1.00	0.86–1.18
	*Women*	171	1.06	0.95–1.17	1.04	0.93–1.16	119	1.25	0.96–1.63
People	***All***	433	1.10	1.00–1.20	1.08	0.98–1.19	345	1.03	0.85–1.25
	*Men*	262	1.13*	1.01–1.26	1.12	0.99–1.25	226	1.02	0.80–1.29
	*Women*	171	1.03	0.87–1.22	1.01	0.85–1.20	119	1.06	0.77–1.45
Things	***All***	433	0.98	0.94–1.03	0.99	0.94–1.03	345	0.99	0.91–1.08
	*Men*	262	0.99	0.94–1.04	0.99	0.94–1.04	226	0.99	0.89–1.09
	*Women*	171	0.97	0.90–1.05	0.97	0.89–1.05	119	1.00	0.85–1.18

Note. N (all)  = 28,778; N (men)  = 15,465; N (women)  = 13,313; Model 1. Cox proportional hazard regression analysis adjusting for age in the time scale with robust standard errors to adjust for correlation within twin pairs, and with sex and smoking as covariates. Model 2. Cox proportional hazards regression analysis stratified by twin pair adjusting for age in the time scale and including sex and smoking as covariates.

*p<.05.

In the analyses stratified by twin pair (see Table 3, Model 2), the associations between occupational complexity with data and people were no longer significant. However, the HR for occupational complexity with data did not change in these analyses. Conversely, the HR for occupational complexity with people dropped from 1.07 to 1.03. When these analyses were further stratified by sex the HR for occupational complexity with data dropped from 1.07 in men in the unstratified analyses to 1.00, but increased from 1.06 to 1.25 in women. The HR for occupational complexity with people decreased in men from 1.13 to 1.02, but increased in women from 1.03 to 1.06. However, the confidence intervals in the stratified and unstratified models overlapped.

Finally, we conducted several sensitivity analyses. We conducted analyses separately for twins born 1886–1925 and 1926–1958 and found results similar to the overall analyses (for twins born 1886–1925,: HR = 1.07, CI = 1.00–1.14, for complexity with data-all; HR = 1.09, CI = 1.00–1.18, for complexity with data-men only; HR = 1.18, CI = 1.03–1.34, complexity with people-men only; for twins born 1926–1958: HR = 1.09, CI = 0.98–1.21, *p*>0.05, for complexity with data-all; complexity with data-men only, HR = 1.05, CI = 0.93–1.20, *p*>0.05; HR = 1.06, CI = 0.87–1.28, *p*>0.05 for complexity with people-men only.

We also examined the effect of education (basic education vs. higher than basic) as a covariate, exploring education as a proxy for those more likely to come into contact with the health care system. The HRs were similar as those from the original analyses, although the confidence intervals widened, reducing the statistical significance of these results (See Table 3).

We excluded people who were in the study less than 15 years to reduce possible bias by reverse causation (n = 67); complexity with data-all, HR = 1.09, 95% CI = 1.03–1.16; complexity with data-men only, HR = 1.13, 95% CI = 1.06–1.20, and complexity with people-men only, HR = 1.16, 95% CI = 1.02–1.33. We also excluded people who were solely identified through the CDR (n = 34) to eliminate any possible differential bias between individual diagnosed while alive and those diagnosed at death; complexity with data-all, HR = 1.08, 95% CI = 1.02–1.14; complexity with data-men only, HR = 1.08, 95% CI = 1.01–1.16, and complexity with people-men only, HR = 1.07, 95% CI = 1.00–1.15.

## Discussion

Several studies have shown a relationship between education or high status occupations and higher risk of PD [4], [9]. We extend this research by examining the association between occupational complexity and risk of Parkinson's disease, presuming that individual dimensions of occupational complexity, which reflect education and occupational status to some extent, may shed light on the previous results by examining cognitive effort required at work. We found that higher occupational complexity with data and people were associated with increased risk of PD which appeared particularly high for higher complexity of work with people among men. Occupational complexity with things was unrelated to PD, probably due to its low reliability [15] or its inability to capture occupational characteristics relevant to risk of neurodegenerative diseases such as dementia [8] and PD.

Analyses stratified by twin pairs yielded non-significant associations, although the HR estimates were not obviously attenuated. Therefore familial factors do not appear to explain our results. However, twin-stratified analyses restricted to men suggested potentially greater familial influence in men. Thus, given the difference between the non-stratified and stratified results, familial factors such as personality and other factors affecting occupation selection, can be presumed to partially underlie the reported findings. Personality figures prominently within the occupational complexity conceptual framework [11] as a predisposing factor to seek occupations of certain type and level of complexity, therefore we do not assume a causative relationship between occupational complexity and risk of PD.

Many of the occupations shown to increase the risk for developing PD by previous research [4], such as business, healthcare, teaching, religious or legal occupations, would be coded as high in occupational complexity with data and people. Although not entirely consistent, epidemiological evidence indicates a pattern in which certain pre-morbid personality traits such as being industrious, punctual, inflexible, ambitious, orderly, and risk avoidant are common among individuals who develop PD [22]. Occupations that involve high occupational complexity with data and people are likely to be sought by someone who meets at least some of these characteristics. Alterations in the dopaminergic pathway [23], [24], whereby certain personality types exhibit dopamine deficiency, offers another potential underlying mechanism for our observed association between occupational complexity and PD. However, not all research supports the hypothesized relationship between PD and personality [25]. The interaction of personality and occupation in relation to PD risk deserves further attention.

Occupations requiring higher complexity with data and people may also involve greater levels of certain types of stress, such as interpersonal conflict associated with mentoring [26] in jobs such as managerial positions or professors [27]. Elevation of stress levels can cause increased levels of glutamate [28], which has been implicated in PD [29].

Limitations of the study include that occupation was only measured at one time point instead of over time. However, substantial job mobility could not be expected in this cohort [30]. Additionally, register-based PD diagnoses do not identify incident cases of PD by strict definition; however, they are a good approximation and there were only 34 cases identified solely by the CDR, and removing them from the analyses did not change the results. Registers have a PPV of 70% and it is highly unlikely that any misdiagnosis is differential by complexity score. In addition, there could be concerns about reverse causation bias, in which having preclinical signs of the disease may have led to different occupational choices; however, our sensitivity analyses which excluded people who were in the study less than 15 years suggested that any bias was negligible.

Another limitation is the potential for medical surveillance bias, in which individuals with higher education or higher complexity occupations may be more likely to seek medical care. As there is a universal health care system in Sweden with equal access to health care across the population, there is a reduced pro-rich bias in obtaining healthcare [31]. Further, we controlled for education as a proxy for those more likely to come into contact with the health care system, but these results were similar to the main results (although statistical significance was reduced). These results indicate that occupational complexity contributes to PD risk beyond the effects of education. Finally, PD is chronically progressive and causes quite notable and disabling symptoms, minimizing opportunity for surveillance bias.

Strengths of the study include the combined use of questionnaire data with comprehensive record of PD diagnoses from the national health registers, with the questionnaire allowing for the assessment of smoking and education. Further, using the STR allowed us to control for the effect of genetic and early life factors using the twin pair analysis.

In conclusion, by investigating individual dimensions of occupational complexity as a risk factor for PD, we provide a novel way of exploring previously reported associations between education, occupation, and risk of PD. People with an occupation high in complexity of work with data or people may be more likely to develop PD, with the increase in risk about even for these dimensions, albeit somewhat higher for complexity of work with people in men. In addition, we provide some evidence that familial factors, such as personality and other factors affecting occupational selection, may play a role in these results, suggesting that the underlying mechanisms may be inherent, at least in men. Further work to uncover the possible mechanisms of this association may be imperative for the success of any future intervention.
